# Low-density lipoprotein cholesterol, C-reactive protein, and lipoprotein(a) universal one-time screening in primary prevention: the EPIC-Norfolk study

**DOI:** 10.1093/eurheartj/ehaf209

**Published:** 2025-04-01

**Authors:** Jordan M Kraaijenhof, Nick S Nurmohamed, Ask T Nordestgaard, Laurens F Reeskamp, Erik S G Stroes, G Kees Hovingh, S Matthijs Boekholdt, Paul M Ridker

**Affiliations:** Department of Vascular Medicine, Amsterdam Cardiovascular Sciences, Amsterdam University Medical Centers, University of Amsterdam, Amsterdam, The Netherlands; Department of Vascular Medicine, Amsterdam Cardiovascular Sciences, Amsterdam University Medical Centers, University of Amsterdam, Amsterdam, The Netherlands; Department of Cardiology, Amsterdam Cardiovascular Sciences, Amsterdam University Medical Centers, Vrije Universiteit Amsterdam, Amsterdam, The Netherlands; Department of Clinical Biochemistry, Copenhagen University Hospital—Herlev and Gentofte, Herlev, Denmark; Department of Vascular Medicine, Amsterdam Cardiovascular Sciences, Amsterdam University Medical Centers, University of Amsterdam, Amsterdam, The Netherlands; Department of Vascular Medicine, Amsterdam Cardiovascular Sciences, Amsterdam University Medical Centers, University of Amsterdam, Amsterdam, The Netherlands; Department of Vascular Medicine, Amsterdam Cardiovascular Sciences, Amsterdam University Medical Centers, University of Amsterdam, Amsterdam, The Netherlands; Department of Cardiology, Amsterdam Cardiovascular Sciences, Amsterdam University Medical Centers, Vrije Universiteit Amsterdam, Amsterdam, The Netherlands; Divisions of Preventive Medicine and Cardiovascular Diseases, Brigham and Women’s Hospital, 900 Commonwealth Ave, Boston, MA 02215, USA

**Keywords:** Atherosclerotic cardiovascular disease, Primary prevention, LDL cholesterol, High-sensitivity C-reactive protein, Lipoprotein(a)

## Abstract

**Background and Aims:**

Recent data from a large American cohort of women strongly support universal one-time screening for LDL cholesterol, high-sensitivity C-reactive protein (hsCRP), and lipoprotein(a) [Lp(a)] in primary prevention. This study addresses the validity and generalizability of this novel primary prevention strategy in a large prospective European cohort of initially healthy men and women.

**Methods:**

Plasma levels of LDL cholesterol, hsCRP, and Lp(a) were measured at study entry in 17 087 participants from the EPIC-Norfolk study who were subsequently followed over a period of 20 years for major adverse cardiovascular events (MACEs). Competing risk- and multivariable-adjusted hazard ratios (HRs) and 95% confidence intervals (CIs) for incident MACE across quintiles of each biomarker and sought evidence of independent as well as additive effects over time were calculated.

**Results:**

During the 20-year follow-up, a total of 3249 MACEs occurred. Increasing quintiles of baseline LDL cholesterol, hsCRP, and Lp(a) all predicted 20-year risks; the multivariable-adjusted HRs in a comparison of the top to bottom quintile were 1.78 (95% CI: 1.57–2.00) for LDL cholesterol, 1.55 (95% CI: 1.37–1.74) for hsCRP, and 1.19 (95% CI: 1.07–1.33) for Lp(a). Compared with individuals with no biomarker elevations, the multivariable-adjusted HRs for incident MACE were 1.33, 1.68, and 2.41 for those with one, two, or three biomarkers in the top quintile, respectively (all *P* < .001). Each biomarker demonstrated independent contributions to overall risk and findings were consistent in analyses stratified by sex.

**Conclusions:**

A single combined measure of LDL cholesterol, hsCRP, and Lp(a) among initially healthy European men and women was predictive of incident MACE during a 20-year period. These data replicate findings from a recent American cohort and strongly support universal screening for all three biomarkers in primary prevention.

## Structured Graphical Abstract

**Structured Graphical Abstract:**
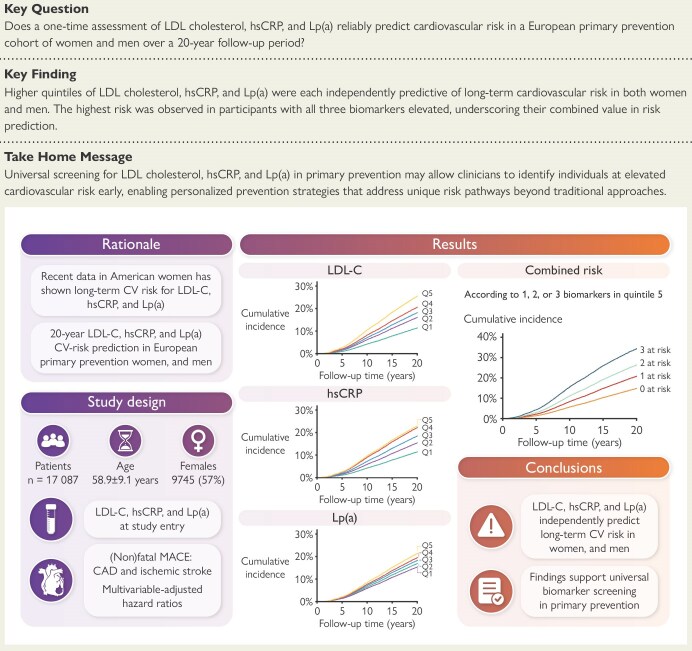

**See the editorial comment for this article ‘Atherosclerosis prediction: three pathways, stronger insights’, by V.Z. Rocha, https://doi.org/10.1093/eurheartj/ehaf396.**

## Introduction

While risk algorithms and imaging tests can inform preventive cardiologists about whom to treat, modifiable blood-based biomarkers can inform physicians about what to treat with. Recently, in the prospective Women’s Health Study (WHS) cohort of 27 939 initially healthy American women, a single baseline measure of three widely available modifiable risk markers—LDL cholesterol, high-sensitivity C-reactive protein (hsCRP), and lipoprotein(a) [Lp(a)]—strongly predicted future cardiovascular events over a 30-year follow-up period.^1^ In that study, each of these three biomarkers, both independently and in unison, predicted unique patterns of risk for individual patients that would otherwise have been missed using traditional prediction algorithms, a highly relevant issue for choice of therapeutics as each of these biomarkers represents a different modifiable pathway leading to atherosclerosis. As such, these data have raised the hypothesis that universal screening for LDL cholesterol, hsCRP, and Lp(a) should be done at least once in all adult patients.^2^ To date, based in part on contemporary secondary prevention data among those taking and not taking statin therapy,^3–5^ European Society guidelines have endorsed widespread screening for hsCRP along with LDL cholesterol among those with chronic coronary syndromes^6^ and one-time screening for Lp(a) has entered primary prevention guidelines based upon robust epidemiologic data.^7–9^ However, universal one-time screening for all three biomarkers has yet to be fully incorporated into guidelines for those without overt evidence of cardiovascular disease.

From a policy perspective, moving universal one-time screening for LDL cholesterol, hsCRP, and Lp(a) into primary prevention as strongly suggested by the WHS requires external replication for consistency, validity, and generalizability. We therefore sought to determine whether the predictive value of LDL cholesterol, hsCRP, and Lp(a), both individually and in combination, extends to a European primary prevention population that also includes men. Towards that end, we report here the association of these three biomarkers with risks of future atherosclerotic events in the prospective Investigation into Cancer and Nutrition (EPIC)-Norfolk study which included 17 087 initially healthy men and women who had each biomarker evaluated once at study entry and who have subsequently been followed over a period of 20 years.^10,11^

## Methods

### Study population

The prospective EPIC-Norfolk study recruited men and women aged 40–79 through general practices in Norfolk, UK, between 1993 and 1997.^10^ Participants completed a detailed health and lifestyle questionnaire, had additional information collected by trained nurses during clinical visits, had high response rates,^11^ and are broadly representative of the British population at that time.^10^

The current analysis was conducted with the data from 17 087 primary prevention EPIC-Norfolk participants who had measures of lipid levels, hsCRP, and Lp(a) available from a blood sample obtained at study entry and who did not have suffered from a major adverse cardiovascular event (MACE) prior to enrolment. Participants were followed up to 25 years, with data available through 31 March 2016. The study received ethical approval from the Norwich District Health Authority Ethics Committee and was conducted in accordance with the Declaration of Helsinki, and all participants provided written informed consent.

### Laboratory measurements

Non-fasting blood samples were obtained at baseline and were either processed immediately at the Department of Clinical Biochemistry, University of Cambridge, or stored at −80°C for future analysis. Plasma levels of total cholesterol, HDL cholesterol, and triglycerides were measured from fresh samples using a RA 1000 auto analyser (Bayer Diagnostics, Basingstoke, UK); LDL cholesterol levels were calculated using the Friedewald formula based on these measurements. hsCRP levels were analysed in all participants with available frozen baseline serum samples using a high-sensitivity Olympus AU640 Chemistry Immuno Analyser (Olympus Diagnostics, Watford, UK).^12,13^ The Lp(a) concentrations were assessed using an isoform-independent immunoturbidimetric assay, employing polyclonal antibodies targeting epitopes of apolipoprotein(a) (Denka Seiken, Coventry, UK).^14^

### Study exposures

The primary objective was to assess the relationship between LDL cholesterol, hsCRP, and Lp(a) and cardiovascular event risk in women and men within the prospective EPIC-Norfolk study. We analysed the impact of each biomarker independently and assessed their combined effect on cardiovascular events. The cardiovascular outcome was a composite of first MACE, defined as fatal or non-fatal coronary artery disease (ICD-10 codes: I20–I25) and fatal or non-fatal ischaemic stroke (ICD-10: I63) consistent with previous EPIC-Norfolk definitions.^12^ Major adverse cardiovascular events were documented during follow-up if participants were hospitalized or died with coronary artery disease or ischaemic stroke as the primary cause. Hospital admissions were tracked through the ENCORE system using National Health Service numbers, ensuring comprehensive capture of hospitalizations.

Study covariates included age, sex, diabetes mellitus, systolic blood pressure, and current smoking. Diabetes mellitus was defined as the use of diabetes medication or a glycated haemoglobin level of 48 mmol/mol (6.5%) or higher. Systolic blood pressure was recorded in mmHg, and smoking status was determined through self-reported questionnaire.

### Statistical analysis

Normally distributed data are presented as mean [standard deviation (SD)], while non-normally distributed data are expressed as median ± interquartile range (IQR). Categorical data are shown as absolute numbers and percentages. Demographic, clinical, and biochemical characteristics are reported for the entire cohort, by biomarker quintiles, and stratified by sex. Spearman’s correlation coefficients were calculated between LDL cholesterol, hsCRP, and Lp(a). Cause-specific Cox regression models were used which accounted for death from other causes as a competing risk across two models. The first model adjusted for age and sex, while the second model included additional adjustments for diabetes mellitus, systolic blood pressure, current smoking, and the presence of the other two biomarkers. We applied sub-distribution hazard models by Fine and Gray^15^ for sensitivity analyses.

For the primary analysis, participants were grouped into quintiles based on biomarker levels, with quintile 1 representing the lowest and quintile 5 the highest concentrations. Hazard ratios (HRs) for cardiovascular events were calculated by comparing quintiles 2 through 5 against quintile 1 as the reference. Associations between each biomarker and cardiovascular events were also assessed both across increasing quintiles and per SD increase in plasma levels, with biomarkers logarithmically transformed for the SD analysis. To evaluate the combined impact of all three biomarkers, participants were categorized as having zero, one, two, or three biomarkers in quintile 5, with the risk calculated for one, two, or three biomarkers using zero as the reference group. Linear trend tests were conducted for all analyses, including those across quintiles and for comparisons of none, one, two, or three elevated biomarkers. All analyses were stratified by sex, using sex-specific biomarker quintiles, to examine potential risk differences between women and men.

Statistical significance was defined as a *P* < .05, and all analyses were conducted using RStudio version 4.3.2 (R Foundation, Vienna, Austria).

## Results

Baseline characteristics of the 17 087 primary prevention EPIC-Norfolk participants evaluated are presented in Supplementary data online, *Table S1*, including stratification by sex. Overall, the mean age was 59.0 years, 11.3% were current smokers, 2.9% had diabetes mellitus, and 16.3% had hypertension with 1.1% using lipid-lowering therapy. Baseline plasma levels of LDL cholesterol, hsCRP, and Lp(a) were 4.0 ± 1.0 mmol/L, 1.5 (0.7–3.2) mg/L, and 11 (6–27) mg/dL, respectively. *Table 1* provides the distributions of these characteristics according to increasing quintiles of LDL cholesterol, hsCRP, and Lp(a). The Spearman correlation coefficient between LDL cholesterol and hsCRP was 0.09, between LDL cholesterol and Lp(a) was 0.19, and between hsCRP and Lp(a) was 0.04.

**Table 1:** Baseline characteristics of 17 087 primary prevention EPIC-Norfolk participants according to quintiles of LDL cholesterol, high-sensitivity C-reactive protein, and lipoprotein(a) evaluated at study entry

Variable	Quintile 1	Quintile 2	Quintile 3	Quintile 4	Quintile 5
LDL cholesterol					
Range of plasma levels, mmol/L	<3.1	3.1 to <3.7	3.7 to <4.2	4.2 to <4.8	≥4.8
Age, years [mean (SD)]	56.1 (9.2)	57.7 (9.2)	59.3 (8.9)	59.9 (8.8)	61.6 (8.6)
Female sex, *n* (%)	2040 (59.2)	1865 (54.6)	1817 (53.3)	1882 (54.9)	2141 (63.1)
Hypertension, *n* (%)	478 (13.9)	514 (15.0)	553 (16.2)	564 (16.5)	676 (19.9)
Diabetes mellitus, *n* (%)	114 (3.3)	118 (3.5)	98 (2.9)	84 (2.5)	83 (2.4)
Current smoker, *n* (%)	379 (11.1)	377 (11.1)	391 (11.6)	376 (11.1)	394 (11.7)
High-sensitivity C-reactive protein					
Range of plasma levels, mg/L	<0.7	0.7 to <1.2	1.2 to <2.1	2.1 to <3.9	≥4.0
Age, years [mean (SD)]	55.3 (8.6)	58.2 (9.1)	59.6 (9.0)	60.7 (8.9)	61.2 (8.8)
Female sex, *n* (%)	2094 (55.9)	1806 (56.6)	1970 (55.5)	1837 (57.2)	2038 (60.2)
Hypertension, *n* (%)	319 (8.5)	387 (12.1)	562 (15.8)	646 (20.1)	871 (25.7)
Diabetes mellitus, *n* (%)	50 (1.3)	55 (1.7)	105 (3.0)	104 (3.2)	183 (5.4)
Current smoker, *n* (%)	324 (8.7)	289 (9.1)	364 (10.3)	399 (12.5)	541 (16.1)
Lipoprotein(a)					
Range of plasma levels, mg/dL	<6	6 to <9	9 to <16	16 to <36	≥36
Age, years [mean (SD)]	58.2 (9.1)	58.3 (9.2)	59.2 (9.2)	59.9 (9.0)	59.2 (9.1)
Female sex, *n* (%)	1911 (55.9)	1934 (56.6)	1943 (56.8)	1964 (57.5)	1993 (58.3)
Hypertension, *n* (%)	540 (15.8)	502 (14.7)	519 (15.2)	649 (19.0)	575 (16.8)
Diabetes mellitus, *n* (%)	106 (3.1)	102 (3.0)	99 (2.9)	89 (2.6)	101 (3.0)
Current smoker, *n* (%)	369 (10.9)	405 (12.0)	408 (12.0)	369 (10.9)	366 (10.8)

### Association of LDL cholesterol, high-sensitivity C-reactive protein, and lipoprotein(a) with cardiovascular disease events

**Figure 1:**
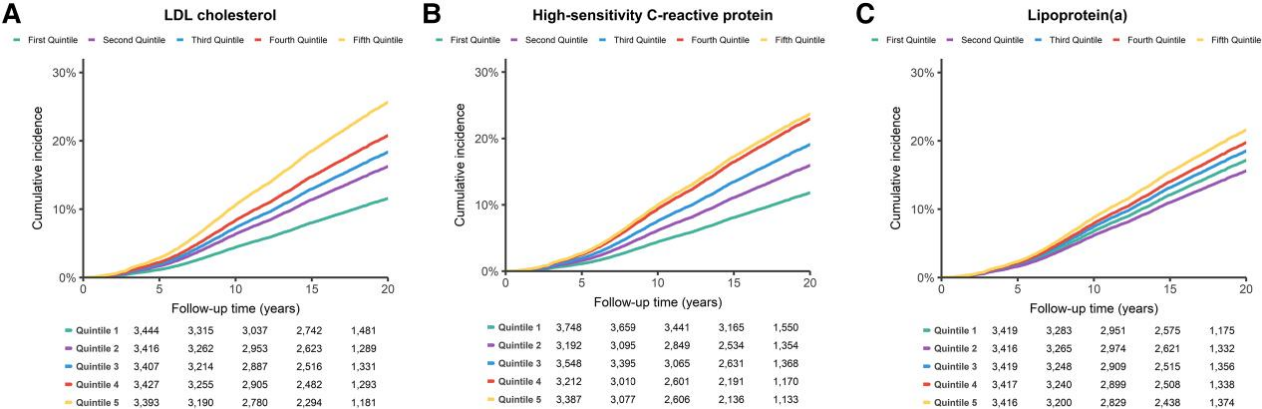
Cumulative incidence for major adverse cardiovascular events of LDL cholesterol, high-sensitivity C-reactive protein, and lipoprotein(a). Cumulative incidence of increasing quintile baseline levels of (*A*) LDL cholesterol, (*B*) high-sensitivity C-reactive protein, and (*C*) lipoprotein(a) for (non)fatal major adverse cardiovascular events, adjusted for death from other causes as a competing risk

Over a median follow-up of 20.5 (IQR: 19.6–21.5) years, a total of 3249 first MACEs were recorded, including 1755 events in the 9745 (18.0%) female participants and 1494 events in the 7342 (20.3%) male participants. The age- and sex-adjusted HRs for MACE comparing the highest quintile (quintile 5) with the lowest (quintile 1) were 1.84 [95% confidence interval (CI): 1.64–2.08] for LDL cholesterol, 1.75 (95% CI: 1.56–1.97) for hsCRP, and 1.28 (95% CI: 1.15–1.42) for Lp(a) (*Table 2*). Following further adjustments in the multivariable model, the HRs were 1.78 (95% CI: 1.57–2.00) for LDL cholesterol, 1.55 (95% CI: 1.37–1.74) for hsCRP, and 1.19 (95% CI: 1.07–1.33) for Lp(a) (*Table 2* and *Figure 1*). For each quintile increase, multivariable-adjusted HRs were 1.14 (95% CI: 1.11–1.17) for LDL cholesterol, 1.12 (95% CI: 1.09–1.15) for hsCRP, and 1.05 (95% CI: 1.02–1.08) for Lp(a). Every standard deviation increase in log-transformed biomarker levels was associated with multivariable-adjusted HRs of 1.14 (95% CI: 1.10–1.18) for LDL cholesterol, 1.07 (95% CI: 1.04–1.09) for hsCRP, and 1.07 (95% CI: 1.04–1.11) for Lp(a) (see Supplementary data online, *Table S2*).

**Table 2:** Hazard ratios for major adverse cardiovascular disease events according to increasing levels of LDL cholesterol, high-sensitivity C-reactive protein, and lipoprotein(a) evaluated at study entry

Variable	Quintile 1	Quintile 2	Quintile 3	Quintile 4	Quintile 5	Per quintile
LDL cholesterol						
MACEs/participants	406/3444	567/3416	640/3407	736/3427	900/3393	
Age- and sex-adjusted hazard ratio	1.0 (reference)	1.26 (1.11, 1.44)	1.33 (1.17, 1.51)	1.49 (1.32, 1.69)	1.84 (1.64, 2.08)	1.15 (1.12, 1.18)
Multivariable-adjusted hazard ratio	1.0 (reference)	1.24 (1.09, 1.41)	1.31 (1.15, 1.48)	1.49 (1.32, 1.68)	1.78 (1.57, 2.00)	1.14 (1.11, 1.17)
High-sensitivity C-reactive protein						
MACEs/participants	456/3748	529/3192	698/3548	754/3212	812/3387	
Age- and sex-adjusted hazard ratio	1.0 (reference)	1.15 (1.02, 1.31)	1.30 (1.16, 1.47)	1.60 (1.42, 1.80)	1.75 (1.56, 1.97)	1.16 (1.13, 1.19)
Multivariable-adjusted hazard ratio	1.0 (reference)	1.11 (0.97, 1.25)	1.20 (1.06, 1.35)	1.44 (1.27, 1.62)	1.55 (1.37, 1.74)	1.12 (1.09, 1.15)
Lipoprotein(a)						
MACEs/participants	596/3419	547/3416	652/3419	694/3417	760/3416	
Age- and sex-adjusted hazard ratio	1.0 (reference)	0.90 (0.80, 1.01)	1.04 (0.93, 1.16)	1.04 (0.93, 1.16)	1.28 (1.15, 1.42)	1.07 (1.04, 1.10)
Multivariable-adjusted hazard ratio	1.0 (reference)	0.88 (0.78, 0.99)	1.00 (0.90, 1.12)	0.97 (0.87, 1.09)	1.19 (1.07, 1.33)	1.05 (1.02, 1.08)

Competing risk-adjusted hazard ratios are shown for both age- and sex-adjusted models, as well as multivariable-adjusted models accounting for current smoking, diabetes, and systolic blood pressure and the other two biomarkers.

**Figure 2:**
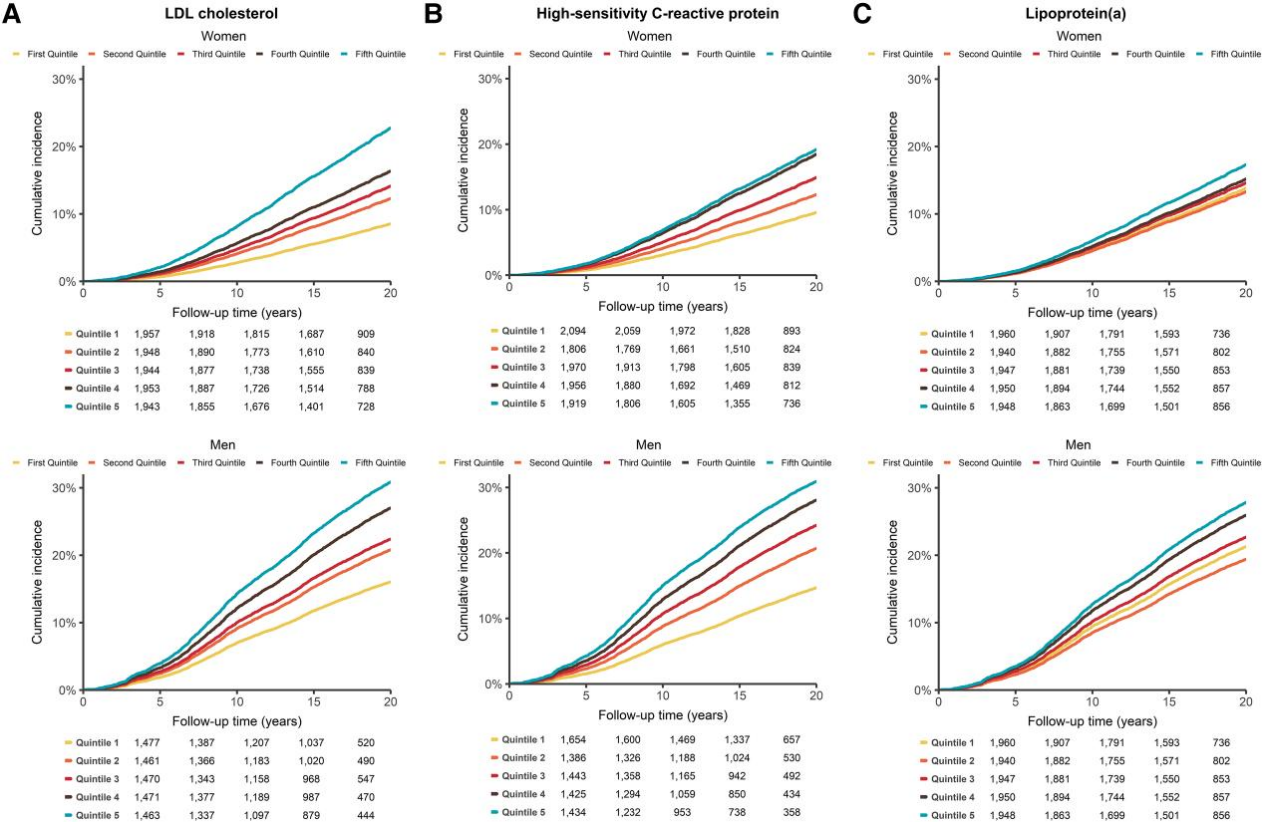
Sex-stratified cumulative incidence for major adverse cardiovascular events of LDL cholesterol, high-sensitivity C-reactive protein, and lipoprotein(a). Cumulative incidence of increasing quintile baseline levels of (*A*) LDL cholesterol, (*B*) high-sensitivity C-reactive protein, and (*C*) lipoprotein(a) for (non)fatal major adverse cardiovascular events stratified for women (*n* = 9745, upper panel) and men (*n* = 7342, lower panel), using sex-specific biomarker quintiles, adjusted for death from other causes as a competing risk

In sex-stratified analyses, the risk associated with the three biomarkers was generally lower in women than in men (*Table 3*). In multivariable-adjusted models comparing the highest to the lowest quintile, the HR for LDL cholesterol was 1.46 (95% CI: 1.21–1.75) in women and 1.91 (95% CI: 1.63–2.23) in men (*Figure 2A*). For hsCRP, the HR was 1.37 (95% CI: 1.15–1.63) in women compared with 1.75 (95% CI: 1.49–2.06) in men (*Figure 2B*), while for Lp(a), the HR was 1.13 (95% CI: 0.96–1.32) in women and 1.26 (95% CI: 1.09–1.46) in men (*Figure 2C*). Each quintile increase in LDL cholesterol was associated with a HR of 1.09 (95% CI: 1.05–1.14) in women and 1.17 (95% CI: 1.13–1.21) in men. For hsCRP, the HR was 1.10 (95% CI: 1.06–1.15) in women and 1.14 (95% CI: 1.10–1.18) in men, while for Lp(a), the HR was 1.03 (95% CI: 0.99–1.07) in women and 1.07 (95% CI: 1.03–1.11) in men per each quintile increase. All three biomarkers demonstrated significant linear trends across quintiles (*P* < .001), except for Lp(a) in women (*P* = .18). Data for each standard deviation increase are presented in Supplementary data online, *Table S2*. Using Fine–Gray models as sensitivity analysis, the estimated sub-distribution HRs remained consistent for the total cohort and stratified for sex (see Supplementary data online, *Table S3*).

**Table 3:** Sex-stratified hazard ratios for major adverse cardiovascular events according to increasing levels of LDL cholesterol, high-sensitivity C-reactive protein, and lipoprotein(a) evaluated at study entry

Variable	Quintile 1	Quintile 2	Quintile 3	Quintile 4	Quintile 5	Per quintile
LDL cholesterol						
Women—MACEs/participants	178/2040	240/1865	261/1817	319/1882	496/2141	
Multivariable-adjusted hazard ratio	1.0 (reference)	1.13 (0.92, 1.37)	1.11 (0.92, 1.35)	1.18 (0.98, 1.43)	1.46 (1.21, 1.75)	1.09 (1.05, 1.14)
Men—MACEs/participants	241/1477	309/1461	336/1470	410/1471	459/1463	
Multivariable-adjusted hazard ratio	1.0 (reference)	1.26 (1.06, 1.49)	1.35 (1.14, 1.60)	1.66 (1.41, 1.95)	1.91 (1.63, 2.23)	1.17 (1.13, 1.21)
High-sensitivity C-reactive protein						
Women—MACEs/participants	207/2094	232/1806	305/1970	350/1837	400/2038	
Multivariable-adjusted hazard ratio	1.0 (reference)	0.96 (0.80, 1.17)	1.06 (0.88, 1.27)	1.28 (1.07, 1.52)	1.37 (1.15, 1.63)	1.10 (1.06, 1.15)
Men—MACEs/participants	249/1654	297/1386	359/1443	406/1425	444/1434	
Multivariable-adjusted hazard ratio	1.0 (reference)	1.23 (1.04, 1.46)	1.31 (1.11, 1.55)	1.53 (1.30, 1.80)	1.75 (1.49, 2.06)	1.14 (1.10, 1.18)
Lipoprotein(a)						
Women—MACEs/participants	271/1911	262/1934	302/1943	307/1964	352/1993	
Multivariable-adjusted hazard ratio	1.0 (reference)	0.93 (0.79, 1.11)	0.94 (0.80, 1.11)	0.92 (0.78, 1.09)	1.13 (0.96 1.32)	1.03 (0.99, 1.07)
Men—MACEs/participants	316/1473	291/1469	340/1465	388/1467	420/1468	
Multivariable-adjusted hazard ratio	1.0 (reference)	0.87 (0.74, 1.02)	1.01 (0.87, 1.18)	1.05 (0.90, 1.22)	1.26 (1.09, 1.46)	1.07 (1.03, 1.11)

Number of (non)fatal major adverse cardiovascular disease events in women (*n* = 9745) and men (*n* = 7342) and corresponding sex-stratified competing risk-adjusted hazard ratios for LDL cholesterol, high-sensitivity C-reactive protein, and lipoprotein(a) across quintiles, using quintile 1 as the reference. Hazard ratios are shown for both age- and sex-adjusted models, as well as the multivariable-adjusted models accounting for current smoking, diabetes, and systolic blood pressure and the other two biomarkers.

### The combined impact of LDL cholesterol, high-sensitivity C-reactive protein, and lipoprotein (a) on cardiovascular disease risk

**Figure 3:**
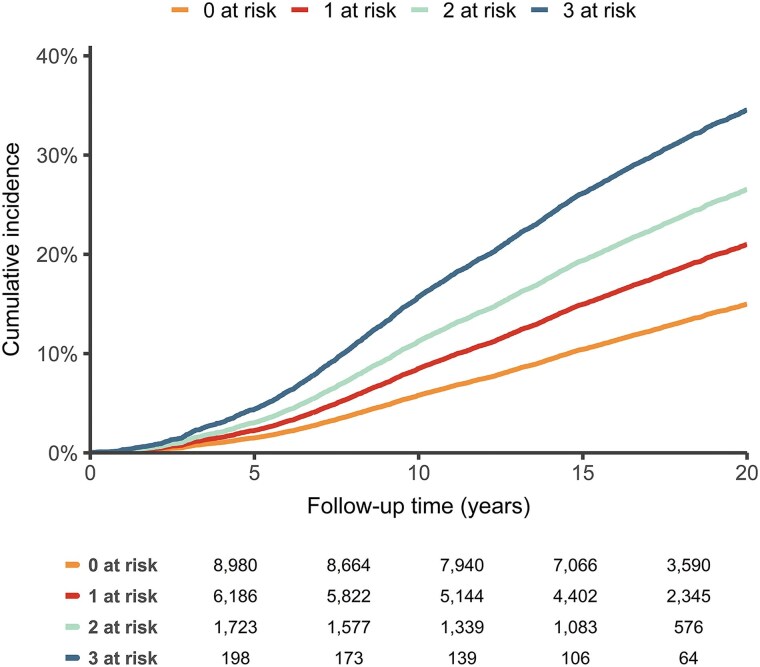
Combined effect of LDL cholesterol, high-sensitivity C-reactive protein, and lipoprotein(a) on the cumulative incidence for major adverse cardiovascular events. Cumulative incidence for (non)fatal major adverse cardiovascular events of the combined effect of LDL cholesterol, high-sensitivity C-reactive protein, and lipoprotein(a) in quintile 5, adjusted for death from other causes as a competing risk. hsCRP, high-sensitivity C-reactive protein; Lp(a), lipoprotein(a)

High levels of LDL cholesterol, hsCRP, and Lp(a) contributed independently to increased cardiovascular risk, with the greatest risk observed when all three biomarkers were in the highest quintile. In the multivariable-adjusted analysis, participants with one biomarker in quintile 5 had a HR for MACE of 1.33 (95% CI: 1.23–1.43) compared with those with no biomarkers in quintile 5 (*Table 4* and *Figure 3*). For participants with two biomarkers in quintile 5, the HR was 1.68 (95% CI: 1.51–1.87), and for those with all three biomarkers in quintile 5, the HR was 2.41 (95% CI: 1.90–3.07).

**Table 4:** Hazard ratios for major adverse cardiovascular events for combined effects of LDL cholesterol, high-sensitivity C-reactive protein, and lipoprotein(a) in quintile 5

	0 biomarkers at risk	1 biomarker at risk	2 biomarkers at risk	3 biomarkers at risk
Age- and sex-adjusted hazard ratio	1.0 (reference)	1.38 (1.28, 1.49)	1.75 (1.58, 1.95)	2.57 (2.03, 3.27)
Multivariable-adjusted hazard ratio	1.0 (reference)	1.33 (1.23, 1.43)	1.68 (1.51, 1.87)	2.41 (1.90, 3.07)
Women—multivariable-adjusted hazard ratio	1.0 (reference)	1.32 (1.18, 1.47)	1.38 (1.17, 1.62)	2.61 (1.93, 3.51)
Men—multivariable-adjusted hazard ratio	1.0 (reference)	1.38 (1.24, 1.53)	2.00 (1.74, 2.30)	1.85 (1.22, 2.81)

Competing risk-adjusted hazard ratios for (non)fatal major adverse cardiovascular events according to the number of biomarkers LDL cholesterol, high-sensitivity C-reactive protein, and lipoprotein(a) in the fifth quintile. Results are shown for both the age- and sex-adjusted model, as well as multivariable-adjusted model accounting for current smoking, diabetes, and systolic blood pressure. Stratified analyses are provided separately for women (*n* = 9745) and men (*n* = 7342), using sex-specific biomarker quintiles.

In sex-stratified analyses, multivariable-adjusted HRs for MACE according to increasing numbers of biomarkers in quintile 5 in women were 1.32 (95% CI: 1.18–1.47) with one biomarker, 1.38 (95% CI: 1.17–1.62) with two biomarkers, and 2.61 (95% CI: 1.93–3.51) with three biomarkers (*Table 4* and Supplementary data online, *Figure S1*). For men, the corresponding HRs were 1.38 (95% CI: 1.24–1.53) with one biomarker, 2.00 (95% CI: 1.74–2.30) with two biomarkers, and 1.85 (95% CI: 1.22–2.81) with three biomarkers in quintile 5. In this latter analysis, however, we note that only 79 of the 7342 men (1.1%) in the EPIC-Norfolk cohort had all three biomarkers in quintile 5 at baseline, thus limiting statistical power. The combined impact demonstrated a significant linear trend (*P* < .001) in the total cohort and within sex-stratified analyses. Results of the Fine–Gray models are shown in Supplementary data online, *Table S4*.

## Discussion

Among 17 078 initially healthy men and women participating in the prospective EPIC-Norfolk study, a one-time baseline screening for LDL cholesterol, hsCRP, and Lp(a) predicted cardiovascular risk over a 20-year follow-up. Across the cohort, increasing quintiles of each biomarker were associated with elevated cardiovascular risk, with the greatest risk discrimination observed when information from all three biomarkers was combined. While the predictive utility of these biomarkers was similar between men and women, absolute risks were larger in men (*Structured Graphical Abstract*).

The present data from the UK provide strong external replication for the concept of universal one-time screening for LDL cholesterol, hsCRP, and Lp(a) in the setting of primary prevention as recently described in a large cohort of American women.^1^ Moreover, as the current data include men, these data extend the generalizability of this novel primary prevention screening strategy.

The similarity of findings between the US cohort and the current UK cohort is striking and provides strong evidence for validity. For example, among women in the UK data, the multivariable-adjusted HR per quintile of LDL cholesterol was 1.09 (95% CI: 1.05–1.14) as compared with a multivariable-adjusted HR per quintile of 1.09 (95% CI: 1.07–1.12) among American women. Similarly, among women in the UK data, the multivariable-adjusted HR per quintile of hsCRP was 1.10 (95% CI: 1.06–1.15) as compared with a multivariable-adjusted HR per quintile of 1.14 (95% CI: 1.12–1.17) among American women. Moreover, in the combined analyses, among women in the UK who had elevated levels of all three biomarkers, the multivariable-adjusted HR compared with women with no elevated biomarkers was 2.61 (95% CI: 1.93–3.51), again fully consistent with the comparable finding among American [multivariable-adjusted HR 2.63 (95% CI: 2.16–3.19)]. Collectively, these results demonstrate that the clinical utility of a one-time screening in primary prevention for LDL cholesterol, hsCRP, and Lp(a) is highly consistent in the USA and the UK. These data are particularly important for women who are frequently underdiagnosed and undertreated for cardiovascular disease.^16,17^

The current findings highlight multiple opportunities for improving primary prevention. First, like the American data which focused on 30-year risks, the current data address 20-year risks, far longer than traditional 10-year risk estimates derived from traditional guideline algorithms. As cardiovascular disease is a lifetime disorder where early prevention achieves the greatest gains, these data reinforce the value and clinical utility of early life detection of risk.

Second, as also shown in the American data, the fact that a single random blood measure of hsCRP provided long-term risk estimates comparable with that of LDL cholesterol and greater than that of Lp(a) should dispense concerns occasionally voiced by some clinicians that variability in hsCRP limits its prognostic value. If anything, as variability is a bias towards the null, these data underscore the unappreciated clinical and pathophysiologic importance of inflammation in the early development of vascular disease.

Third, we further believe these data have implications for both lifestyle and pharmacologic interventions designed to reduce lifelong cardiovascular event rates. European Society guidelines for dietary discretion, smoking cessation, and daily exercise consistently indicate that early intervention provides greatest benefit.^18^ While levels of Lp(a) are generally not influenced by lifestyle changes, these interventions both reduce risk and lower levels of LDL cholesterol and hsCRP.

Finally, one-time simultaneous evaluation of LDL cholesterol, hsCRP, and Lp(a) is also likely to assist clinicians as they select different agents to address the unique aspects of risk presented by individual patients. In this respect, we believe the ‘one size fits all’ approach to pharmacologic primary prevention is outdated as several novel therapeutics beyond statins are entering clinical practice. As examples, both sodium–glucose co-transporter 2 inhibitors and glucagon-like peptide-1 receptor agonists lower hsCRP and cardiovascular risk; the US Food and Drug Administration has approved low-dose colchicine in high-risk primary prevention; and large-scale trials are ongoing on a global basis evaluating interleukin-6 inhibition as a potential novel method to lower cardiovascular event rates beyond lipid-lowering.^19^ With elevated Lp(a) levels unaffected by traditional lipid-lowering strategies, emerging targeted therapeutics may soon offer clinicians a means to manage this distinctive cardiovascular risk.^8^ As was also observed in the prior American data, our combined effects analyses provide strong long-term evidence that multiple pathways are involved in atherosclerosis and thus that targeted strategies for prevention may well need to differ between individuals.

### Limitations

Despite our many strengths and the external consistency of these data, limitations of our study need consideration. Perhaps most important, the overall prevalence of diabetes, hypertension, and obesity is higher today than between 1993 and 1997 when participants were recruited into EPIC-Norfolk. We suspect, however, that our findings are thus—if anything—even more relevant today. We also lack data on statin initiation during follow-up. However, in the first 10 years of our study, statin use in Europe for primary prevention was minimal and only began to rise in prevalence thereafter but was still relatively low in 2016 when our 20-year follow-up was concluded for analysis. We note, however, that in the recent WHS analysis, where statin initiation was addressed over time, censoring follow-up data at the time of statin initiation had minimal effect on the multivariable-adjusted HRs for the individual biomarkers or their combined impact.^1^ Third, the number of ischaemic stroke events was insufficient to allow separate analyses for stroke and coronary artery disease as done in the prior American study. Further, while our data using combined biomarkers from EPIC-Norfolk replicate earlier work in the US cohort, our gender-specific analyses for combined biomarkers were likely underpowered. Last, the EPIC-Norfolk cohort primarily consists of men and women of white descent, restricting the applicability of our findings to other ethnicities.

## Conclusions

Physicians will not treat what they do not measure. In this prospective study of 17 087 European men and women, we demonstrate that a single one-time simultaneous evaluation of LDL cholesterol, hsCRP, and Lp(a) predicts cardiovascular risk over a subsequent 20-year period. These data both replicate and extend very recent American evidence that a simple biomarker panel can detect unique patterns of risk for many patients that would otherwise be missed using traditional global risk algorithms or commonly used imaging tests. As commercial assays for hsCRP and Lp(a) are standardized, inexpensive, and widely available, we believe the time has come for universal screening of these three biomarkers in primary as well as secondary prevention.

## Supplementary Material

ehaf209_Supplementary_Data

## Declarations

### Data Availability

All data are available from the corresponding author upon reasonable request.

### Pre-registered Clinical Trial Number

None supplied.
