# Supplementary material for: Low-density lipoprotein cholesterol, C-reactive protein, and lipoprotein(a) universal one-time screening in primary prevention: the EPIC-Norfolk study
Source: Eur Heart J. 2025 Apr 1;46(39):3875–84. doi: 10.1093/eurheartj/ehaf209 (PMC12517748; doi:10.1093/eurheartj/ehaf209)
Supplement: ehaf209_Supplementary_Data [file ehaf209_supplementary_data.docx]

**Table S1. Baseline characteristics in the EPIC-Norfolk study.**

| **Characteristics** | **Overall cohort** | **Women** | **Men** |
| --- | --- | --- | --- |
| Number of patients | 17,087 | 9,745 | 7,342 |
| Age, years [mean (SD)] | 58.9 (9.1) | 58.7 (9.2) | 59.2 (9.1) |
| Female sex, N (%) | 9,745 (57.0) | - | - |
| Current smoker, N (%) | 1,917 (11.3) | 1,053 (10.9) | 864 (11.8) |
| Diabetes mellitus, N (%) | 497 (2.9) | 216 (2.2) | 281 (3.8) |
| Hypertension, N (%) | 2,785 (16.3) | 1,685 (17.3) | 1,100 (15.0) |
| Lipid-lowering drug use, N (%) | 181 (1.1) | 114 (1.2) | 67 (0.9) |
| BMI, kg/m^2^ (median [IQR]) | 25.7 [23.6, 28.2] | 25.3 [23.1, 28.2] | 26.1 [24.2, 28.2] |
| SBP, mmHg [mean (SD)] | 134.8 (18.3) | 133.2 (18.7) | 136.9 (17.5) |
| DBP, mmHg [mean (SD)] | 82.1 (11.1) | 80.6 (11.0) | 84.2 (11.0) |
| TC, mmol/L [mean (SD)] | 6.1 (1.1) | 6.3 (1.2) | 6.0 (1.1) |
| LDL-C, mmol/L [mean (SD)] | 4.0 (1.0) | 4.0 (1.11) | 3.9 (1.0) |
| HDL-C, mmol/L [mean (SD)] | 1.4 (0.4) | 1.6 (0.4) | 1.2 (0.3) |
| TG, mmol/L (median [IQR]) | 1.5 [1.1, 2.1] | 1.4 [1.0, 1.9] | 1.7 [1.2, 2.4] |
| Lp(a), mg/dL (median [IQR]) | 11 [6, 27] | 12 [6, 28] | 11 [6, 27] |
| hsCRP, mg/L (median [IQR]) | 1.5 [0.7, 3.2] | 1.5 [0.7, 3.3] | 1.4 [0.7, 3.0] |

Baseline characteristics for the complete cohort and stratified by sex. Normally distributed variables are reported as mean (SD), non-normally distributed variables as median ± interquartile range [IQR], and categorical variables as number (%). BMI: body mass index, SBP: systolic blood pressure, DBP: diastolic blood pressure, TC: total cholesterol, LDL-C: low-density lipoprotein cholesterol, HDL-C: high-density lipoprotein cholesterol, TG: triglycerides, Lp(a): lipoprotein(a), hsCRP: high-sensitivity C-reactive protein.

**Table S2. Hazard ratios for major adverse cardiovascular events per standard deviation of log-transformed LDL-C, hsCRP and Lp(a).**

| **Variable** | **Per SD increase** |
| --- | --- |
| **LDL cholesterol** |  |
| Total cohort | 1.14 (1.10, 1.18) |
| Women | 1.13 (1.07, 1.19) |
| Men | 1.22 (1.16, 1.28) |
| **High-sensitivity C-reactive protein** |  |
| Total cohort | 1.07 (1.04, 1.09) |
| Women | 1.07 (1.04, 1.11) |
| Men | 1.11 (1.07, 1.14) |
| **Lipoprotein(a)** |  |
| Total cohort | 1.07 (1.04, 1.11) |
| Women | 1.06 (1.01, 1.11) |
| Men | 1.10 (1.05, 1.15) |

Competing risk- and multivariable-adjusted hazard ratios for (non)fatal major adverse cardiovascular events of each standard deviation increase (SD) in the natural log-transformed levels of LDL cholesterol, hsCRP, and Lp(a), adjusted for age, sex current smoking, diabetes, systolic blood pressure and the other two biomarkers. Multivariable-adjusted analyses are also provided for women (n=9,745) and men (n=7,342). LDL: low-density lipoprotein, hsCRP: high-sensitivity C-reactive protein, Lp(a): lipoprotein(a).

**Table S3. Fine-Gray subdistribution hazard ratios for MACE across increasing LDL-C, hsCRP and Lp(a) quintile levels.**

| **Variable** | **Quintile 1** | **Quintile 2** | **Quintile 3** | **Quintile 4** | **Quintile 5** | **Per quintile** |
| --- | --- | --- | --- | --- | --- | --- |
| **LDL cholesterol** |  |  |  |  |  |  |
| Total cohort | 1.0 (reference) | 1.27 (1.13, 1.42) | 1.34 (1.19, 1.49) | 1.52 (1.37, 1.70) | 1.79 (1.60, 1.99) | 1.14 (1.12, 1.17) |
| Women | 1.0 (reference) | 1.29 (1.01, 1.44) | 1.20 (1.00, 1.43) | 1.31 (1.10, 1.55) | 1.56 (1.32, 1.84) | 1.11 (1.07, 1.14) |
| Men | 1.0 (reference) | 1.27 (1.10, 1.47) | 1.32 (1.14, 1.52) | 1.64 (1.43, 1.89) | 1.78 (1.55, 2.05) | 1.15 (1.12, 1.18) |
| **High-sensitivity C-reactive protein** |  |  |  |  |  |  |
| Total cohort | 1.0 (reference) | 1.14 (1.01, 1.27) | 1.21 (1.08, 1.34) | 1.35 (1.22, 1.51) | 1.35 (1.21, 1.50) | 1.08 (1.05, 1.10) |
| Women | 1.0 (reference) | 1.10 (0.85, 1.19) | 1.11 (0.95, 1.31) | 1.27 (1.08, 1.49) | 1.28 (1.09, 1.49) | 1.08 (1.04, 1.11) |
| Men | 1.0 (reference) | 1.25 (1.08, 1.46) | 1.31 (1.13, 1.51) | 1.42 (1.23, 1.64) | 1.37 (1.27, 1.69) | 1.09 (1.06, 1.12) |
| **Lipoprotein(a)** |  |  |  |  |  |  |
| Total cohort | 1.0 (reference) | 0.90 (0.81, 1.00) | 1.01 (0.92, 1.11) | 1.00 (0.92, 1.11) | 1.15 (1.04, 1.26) | 1.04 (1.02, 1.06) |
| Women | 1.0 (reference) | 0.92 (0.79, 1.07) | 0.96 (0.83, 1.11) | 0.92 (0.79, 1.06) | 1.06 (0.92 1.22) | 1.01 (0.98, 1.05) |
| Men | 1.0 (reference) | 0.91 (0.79, 1.05) | 1.03 (0.91, 1.18) | 1.10 (0.97, 1.25) | 1.23 (1.08, 1.39) | 1.06 (1.03, 1.09) |

Competing risk- and multivariable-adjusted subdistribution hazard ratios for the total cohort, and stratified for women (n=9,745) and men (n=7,342) calculated with the Fine-Gray model. MACE: major adverse cardiovascular events.

**Table S4. Fine-Gray subdistribution hazard ratios for MACE for the combined impact of LDL-C, hsCRP and Lp(a) in the highest quintile.**

|  | 0 biomarkers at risk | 1 biomarker at risk | 2 biomarkers at risk | 3 biomarkers at risk |
| --- | --- | --- | --- | --- |
| Total cohort | 1.0 (reference) | 1.27 (1.19, 1.36) | 1.48 (1.35, 1.62) | 1.95 (1.61, 2.37) |
| Women | 1.0 (reference) | 1.25 (1.13, 1.38) | 1.30 (1.14, 1.50) | 2.13 (1.70, 2.67) |
| Men | 1.0 (reference) | 1.30 (1.19, 1.42) | 1.66 (1.48, 1.87) | 1.35 (0.95, 1.91) |

Competing risk- and multivariable-adjusted subdistribution hazard ratios for (non)fatal major adverse cardiovascular events according to the number of biomarkers LDL cholesterol, hsCRP and Lp(a) in the fifth quintile, calculated with the Fine-Gray model. Stratified analyses are provided separately for women (n=9,745) and men (n=7,342), using sex-specific biomarker quintiles. LDL: low-density lipoprotein, hsCRP: high-sensitivity C-reactive protein, Lp(a): lipoprotein(a).

**Figure S1. Sex-stratified cumulative incidence for major adverse cardiovascular events according to increasing biomarkers in quintile 5.**


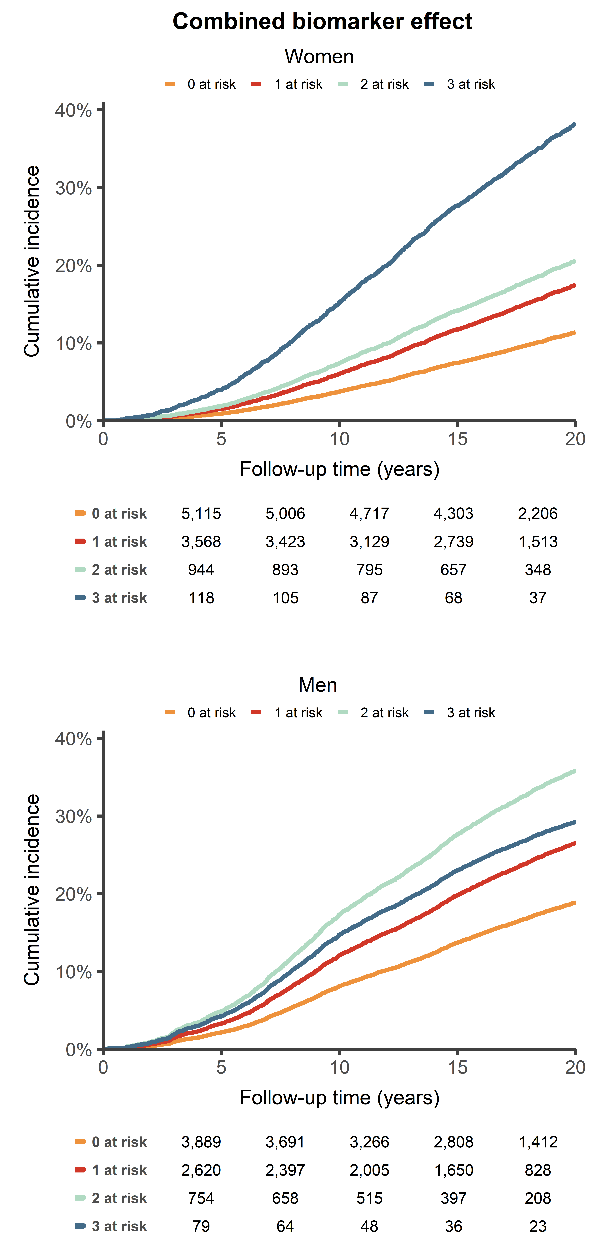


Cumulative incidence for (non)fatal major adverse cardiovascular events of the combined effect of LDL cholesterol, hsCRP, and Lp(a) in women (n=9,745, upper panel) and men (n=7,342, lower panel), according to whether participants had zero, one, two, or three biomarkers in quintile 5, using sex-specific biomarker quintiles, adjusted for death from other causes as a competing risk. LDL: low-density lipoprotein, hsCRP: high-sensitivity C-reactive protein, Lp(a): lipoprotein(a).
